# Illuminating an Invisible Epidemic: A Systemic Review of the Clinical and Economic Benefits of Early Diagnosis and Treatment in Inflammatory Disease and Related Syndromes

**DOI:** 10.3390/jcm8040493

**Published:** 2019-04-11

**Authors:** Lukasz S. Wylezinski, Jamieson D. Gray, Julia B. Polk, Andrew J. Harmata, Charles F. Spurlock

**Affiliations:** 1 Department of Medicine, Vanderbilt University School of Medicine, Nashville, TN 37232, USA; lukasz.wylezinski@iquity.com; 2 IQuity, Inc., Nashville, TN 37203, USA; jamieson.gray@iquity.com (J.D.G.); julia@iquity.com (J.B.P.); drew@iquity.com (A.J.H.)

**Keywords:** multiple sclerosis, Crohn’s disease, ulcerative colitis, fibromyalgia syndrome, irritable bowel syndrome, disease-modifying therapy, diagnostics, direct costs, early diagnosis, inflammation

## Abstract

Healthcare expenditures in the United States are growing at an alarming level with the Centers for Medicare and Medicaid Services (CMS) projecting that they will reach $5.7 trillion per year by 2026. Inflammatory diseases and related syndromes are growing in prevalence among Western societies. This growing population that affects close to 60 million people in the U.S. places a significant burden on the healthcare system. Characterized by relatively slow development, these diseases and syndromes prove challenging to diagnose, leading to delayed treatment against the backdrop of inevitable disability progression. Patients require healthcare attention but are initially hidden from clinician’s view by the seemingly generalized, non-specific symptoms. It is imperative to identify and manage these underlying conditions to slow disease progression and reduce the likelihood that costly comorbidities will develop. Enhanced diagnostic criteria coupled with additional technological innovation to identify inflammatory conditions earlier is necessary and in the best interest of all healthcare stakeholders. The current total cost to the U.S. healthcare system is at least $90B dollars annually. Through unique analysis of financial cost drivers, this review identifies opportunities to improve clinical outcomes and help control these disease-related costs by 20% or more.

## 1. Introduction

Mounting an effective immune response allows mankind to fight off infection and perpetuate survival of the species. However, when the immune system becomes dysregulated, it can cause serious harm and even lead to tissue and organ system destruction in the case of prolonged inflammation [1]. Due to their chronic nature, inflammatory conditions result in increasing patient disability and require lifelong therapy leading to extensive healthcare expense.

Inflammatory disease develops from persistent activation of the immune system that manifests in localized and/or disseminated signs and symptoms throughout the body [1]. Inflammatory diseases exhibit a diverse pathophysiology and affect multiple organ systems, which can lead to widespread damage that is often irreversible [2,3,4]. Initial symptoms can be generic, non-specific, and frequently lead to diagnostic difficulties and delays [5]. In early disease, ruling out syndromes or functional disorders that mimic early inflammatory processes make arriving at a definitive diagnosis of a specific inflammatory disease a challenge. The similarities in manifestation, that are particularly difficult to discern in the early stages of an inflammatory process when physical damage due to inflammation is minimal, often present a challenge to clinicians. They are forced to decipher the cause of non-specific signs and symptoms using laboratory tests or imaging studies that are often inconclusive. This fuels diagnostic confusion and risk of misdiagnosis [6,7].

In current clinical practice, the diagnostic odyssey for patients with inflammatory disease is typically a multi-year process and can require visits with multiple physician specialties as the underlying cause of a patient’s symptoms are investigated. At times, a diagnosis of exclusion is made during this process. This diagnostic methodology presents a difficult situation for both the patient and physician, as the diagnosis rests predominantly on clinical grounds with little to no conclusive evidence from standard laboratory tests or imaging studies [8]. This tactic can result in a lengthy trial and error process, all the while the patient suffers unabated tissue damage. The prolonged period of delayed diagnosis and treatment initiation places substantial burden on patients and can result in increased healthcare costs [9,10]. There is a growing consensus that early intervention is vital for more positive patient outcomes and to curtail the exponential growth of healthcare spending. The ability to shorten the time to an accurate diagnosis and initiate the most appropriate course of treatment is in all stakeholders’ best interest. Early diagnosis and effective treatment of inflammatory diseases ensures the best patient outcomes while lowering financial impact on the healthcare system [11,12,13].

In an effort to quantify the financial impact felt throughout the healthcare system, this work investigated cost drivers associated with inflammatory diseases and related syndromes and calculated how improved clinical efficiencies for diagnosis and treatment of these conditions could reduce the overall financial burden. Four critical cost factors were determined after an extensive review of the literature: (1) treatment, (2) controlling condition progression, (3) avoidance of high-cost events, and (4) promotion of treatment adherence. For the inflammatory diseases and related conditions that we examined, the cost-savings potential across all of these diseases collectively is estimated to be greater than 20%.

This work is among the first comprehensive overviews of prevalence, clinical characteristics, challenges, and financial impacts of four inflammatory diseases and two related syndromes that have an estimated cost of at least $90B annually adjusted to 2018 dollars. This estimate incorporates the lowest cost factor for each condition and multiplies these costs by disease prevalence. Understanding the clinical dilemmas faced during the diagnostic and treatment odyssey, along with the financial impact on a strained U.S. healthcare system, uncovers opportunities for intervention that may lead to enhanced patient outcomes and healthcare cost savings.

## 2. Clinical Manifestation, Diagnosis, and Management of Inflammatory Diseases and Chronic Syndromes

Four well-studied inflammatory diseases that affect different organ systems include multiple sclerosis (MS), ulcerative colitis (UC), Crohn’s disease (CD), and rheumatoid arthritis (RA). These four diseases affect over four million people in the U.S. and cost the healthcare system an aggregated total of over $35B per year in direct costs alone, adjusted to 2018 inflated dollars.

### 2.1. Multiple Sclerosis

Multiple sclerosis is an inflammatory neurodegenerative disease affecting the central nervous system (CNS) that leads to demyelination of neurons [14]. Two and a half million people worldwide are currently living with MS, including an estimated 1 million patients in the U.S. alone (Table 1) [14,15,16]. There are 16,500 new patients diagnosed with MS each year in the U.S., with women being affected three times more often than men [17]. Peak incidence of MS occurs in the third to fourth decade of life and persists throughout the rest of the patient’s lifespan. Patients suffer steadily increasing disability and accumulating healthcare costs [17].

The pathology of MS is characterized by infiltration of auto-reactive T cells, B cells, and other immune mediators into the CNS, culminating in widespread neuroinflammation and neurodegeneration [30]. Neuroinflammation results in patients developing symptoms that include sensory disturbances, visual impairments, muscle weakness, difficulty walking, and problems with coordination [31]. More distinctive symptoms of MS typically occur in a waxing and waning manner, increasing the difficulty of correctly diagnosing the condition. Symptoms usually develop over the course of days and remit over the ensuing weeks or months [32]. The unique nature of some sensory symptoms and the difficulty patients experience in describing such symptoms may result in a misdiagnosis of another condition. For instance, MS may be mistaken as somatic syndrome disorder where patients can present with very similar initial symptoms of fatigue, headache, trembling, insomnia, and cognitive issues [33,34]. Symptoms progress over the course of disease as damage to the CNS accumulates. Furthermore, relapses or episodes of exacerbation of symptoms caused by a demyelinating event occur periodically. A relapse often requires acute treatment and may even lead to hospitalization [35]. The growing consensus is that early diagnosis and treatment are vital to slowing progression of disease and staving off mounting patient disability [3,13].

Clinicians arrive at a diagnosis of MS after evaluating the clinical and laboratory findings required to fulfill the McDonald Criteria [36,37]. Brain and spine magnetic resonance imaging (MRI) studies are performed to document changes in brain lesion dissemination in time and space alongside collection of a detailed patient clinical history and physical examination [36,37]. Additional testing of cerebrospinal fluid-specific oligoclonal bands and visual evoked potentials is also supported by the current McDonald Criteria. Newly developed tools, including analysis of serum proteins and RNA profiles derived from peripheral whole blood, have also been reported as emerging technologies that may support a diagnosis of MS [36,38,39]. A combination of approaches is frequently used by clinician as there are no definitive singular clinical or laboratory findings to rule in or rule out a diagnosis of MS.

In an effort to mitigate disability and slow disease progression, patients are prescribed disease modifying therapies (DMTs) [40]. These medications reduce the frequency and severity of relapses by suppressing the immune system [31]. In MS, DMTs can reduce annualized relapse rates from 17% to 72% versus placebo control [41]. The range of DMT efficacy correlates directly with risk to patients, thus a risk–benefit analysis is recommended prior to selecting a course of treatment, as unpleasant side effects and serious adverse events can be experienced [42]. The potential for adverse side effects underscores the importance of initiating the optimal treatment that takes into account the severity of disease, as well as the negative effects of therapy. Patient input is required, as loss of treatment adherence due to unanticipated or unpleasant side effects risk leaving the patient with uncontrolled MS [43].

Rampant, uncontrolled inflammation can lead to the development of comorbidities that further complicate disease. MS patients have an increased propensity to develop comorbidities such as depression, anxiety, cardiovascular complications, and inflammatory bowel disease [2,44]. These comorbidities can create diagnostic delays, increase the risk of disability progression, and result in more frequent hospitalizations [45]. Previous work has shown that mean diagnostic delay can reach seven years when factoring in complicated cases due to a wide range of associated comorbidities [46]. The development of MS and accumulation of comorbidities, resulting in mental and physical disability, leads to rapid deterioration in patient quality of life [47]. 

MS disease burden pervades all aspects of a patient’s life, affecting both the personal and professional realm. Workplace studies have shown that increased levels of absenteeism (lost working days) and presenteeism (minimal productivity while on the job) are frequently observed in MS patients. Working individuals with MS were absent four times more often than their coworkers [48]. Presenteeism has been shown to approach five hours of lost work per week due to disability associated with MS [49]. These indirect costs, when combined with the direct healthcare expenditures, represent a significant cost-savings potential that could be achieved through early detection and initiation of treatments to minimize disease progression [50].

### 2.2. Ulcerative Colitis and Crohn’s Disease

Inflammatory bowel disease (IBD) is an umbrella term comprising chronic idiopathic inflammatory diseases of the gastrointestinal tract. The two predominant forms of IBD are ulcerative colitis (UC) and Crohn’s disease (CD) [21]. In the U.S., there are over 1.6 million people diagnosed with IBD and more than 65,000 new patients are diagnosed each year (Table 1) [18]. Both UC and CD have similar onset, with peak incidence in the second to fourth decade of life [51]. Disease typically manifests with mild to moderate symptoms affecting the gastrointestinal tract, but may be complicated by extraintestinal symptoms including fever, fatigue, and weight loss. As a group, these extraintestinal symptoms are seen in 25%–40% of all IBD patients [52,53,54]. Almost every organ system can be affected, but symptoms involving the eyes, skin, liver, and joints are considered primary manifestations outside of the gastrointestinal system [52]. 

Typically, primary care physicians will consider multiple pathologies in addition to IBD at the time of patient presentation [55]. An aggregation of patient history, physical examination, laboratory testing, and endoscopy are required for diagnosis of UC or CD [56]. Endoscopic evaluation allows direct visualization and biopsy of the lower gastrointestinal (GI) track mucosa to identify bowel damage. Unfortunately, endoscopic evaluation can be nonspecific if the findings overlap other diseases like infective colitis [57]. Thus, to achieve a high degree of accuracy in diagnosing IBD as well as establishing IBD classification, endoscopic imaging and biopsy are often used in conjunction with other laboratory findings [58]. At best, these approaches can differentiate UC from CD in 85% of patients. Furthermore, distinguishing UC and CD is most difficult early in disease pathogenesis [59,60]. The misdiagnosis rate for IBD, including a change in diagnosis from IBD to a non-IBD diagnosis or vice-versa, is reported to be as high as 23.5% (Table 1) [26]. In certain cases, where it is impossible for the attending physician to reach a final diagnosis of UC or CD, the designation of indeterminate colitis (IC) is used, accounting for up to 15% of IBD patients [61]. Opinions vary on the classification of IC, as some physicians classify it as a distinct autonomic pathoclinical unit with similar etiology to UC or CD, while others use the IC classification as a temporary diagnosis and rely on additional diagnostic testing over time to establish a diagnosis of CD or UC [61]. Newly developed diagnostic strategies, including novel biochemical assays looking for serum proteins or expression markers derived from peripheral whole blood, could provide additional diagnostic information not only to accurately classify UC and CD, but also to assess disease activity and understand when certain therapies are more or less effective in distinct patient populations [60,62,63].

While patients experience a similar diagnostic journey preceding a diagnosis of UC or CD, treatment of these diseases differs significantly. In mild to moderate forms of IBD, treatment for UC utilizes aminosalicylates along with steroids, while enteral nutrition or glucocorticoids are common treatments for CD [56]. In instances of severe disease or in patients refractory to initial treatment, biological immunosuppressive drugs are prescribed in both UC and CD [64]. Selecting the optimal course of treatment for each individual helps to induce remission of disease and reduce the probability of high-risk relapses. Disease remission increases 12%–25% in IBD patient populations receiving pharmacological treatment [65]. However, in approximately 20%–30% of IBD patients receiving conventional treatments, disease progression will eventually necessitate surgical intervention [66,67,68].

Like MS, IBD has a significant impact on patient quality of life. Patients change social and family activities, alter dietary choices, and frequently experience an increased need for third-party caregivers [69]. IBD also negatively impacts workplace performance including increased indirect costs due to lost productivity [70,71]. IBD patients suffer from chronic work disability at a rate that is 17% higher than the general population and take sick leave 10 more days each year compared to others in the workplace [71]. Work disability and sick leave are among the greatest cost drivers when the total indirect costs associated with IBD are calculated [72].

### 2.3. Rheumatoid Arthritis

Rheumatoid arthritis (RA) is characterized by tenderness, swelling, and destruction of synovial joints through erosion of cartilage and bone that can lead to severe disability or premature mortality [73]. There are over 1.6 million people in the U.S. living with RA and this patient group is growing by at least 130,000 new patients each year (Table 1) [19,23]. RA affects women three times more frequently than men and incidence of disease increases with age. The peak onset of adult RA typically occurs within the sixth decade of life [19,74]. Ultimately, joint destruction can lead to loss of physical function, an inability to carry out daily activities, and can result in difficulty maintaining employment [75].

RA presents clinically as persistent synovitis and systemic inflammation. It is also characterized by autoantibody production and release of inflammatory cytokines and chemokines in a large portion of patients [76]. RA is diagnosed after careful evaluation of patient history, physical examination, and selected laboratory testing. The American College of Rheumatology/European League Against Rheumatism 2010 RA classification criteria has been adopted in an effort to focus on features that permit diagnosis at an earlier disease stage rather than waiting for disease defining late-stage characteristics [73]. The criteria are meant to be applied to patients that exhibit synovitis in at least one joint, but it also incorporates laboratory findings to detect antibody production and acute-phase reactants [73]. As with other inflammatory diseases, even the best laboratory tests for RA cannot be used as a singular test to establish a diagnosis as they often fail to capture the entire disease population. For example, certain autoantibody laboratory tests recognize levels of anti-citrullinated peptides, but are only effective in a portion of the patient population, as up to 20% of patients with RA will never test positive for autoantibodies pre or post diagnosis [77]. Erythrocyte sedimentation rates (ESR) and C-reactive protein levels are generally correlated with radiographic findings of joint damage [73]. These measurements, however, can be elevated by other infectious and inflammatory processes that are not specific to RA, or can be altered, in the case of ESR, by acute phase reactants from malignancy, tissue injury, or trauma [78]. Thus, while these tools may be helpful to clinicians during the diagnostic process, there remains no single test that can be used to diagnose RA. 

The predominant treatments for RA are disease-modifying anti-rheumatic drugs (DMARDs), which include a wide range of compounds that can be prescribed alone or in conjunction with either biologics and/or glucocorticoids [76]. The objective is to begin treatment with DMARDs as soon as possible in hopes of tightly controlling disease activity and progression with the ultimate goal being to achieve a state of clinical remission. When selecting treatment, factors apart from disease activity such as progression of structural damage, comorbidities, and safety concerns, should be recognized [79]. Although biologics have revolutionized the field of RA treatment, their overwhelming cost, increased risk of causing serious infections, and limited availability make them less accessible to a majority of the population [80,81]. When started early in naïve RA patients, combination therapy of various non-biologic conventional DMARDs has been shown to have similar efficacy as biologics [82]. Early intervention presents clinicians with an opportunity to arrest progression of disease through use of cost-effective, non-biologic therapies and to reduce the direct costs associated with RA disease management [82]. When left untreated or unresponsive to therapy, RA leads to persistent joint inflammation, progressive joint damage, and continuing functional decline, in addition to the development of serious comorbidities [83]. 

### 2.4. Chronic Syndromes

A number of functional disorders are often confused with or discovered alongside an inflammatory disease diagnosis. The co-occurrence of these chronic syndromes hints at a possible link but the precise biological or environmental disturbance that results in either disease class remains unclear [84,85]. Two examples of well-documented functional disorders with high prevalence are irritable bowel syndrome (IBS) and fibromyalgia syndrome (FMS). MS patients are known to develop IBS in 10% of cases and up to 20% of patients with RA have been shown to also fulfill diagnostic criteria for FMS [2,44,86,87,88,89,90]. Management of these conditions accounts for less direct costs when compared to inflammatory diseases, but higher prevalence among these syndromes produces a cost burden that is larger than most inflammatory disorders [20,91]. 

### 2.5. Irritable Bowel Syndrome

Irritable bowel syndrome (IBS) is characterized by chronic or recurrent abdominal pain and bloating with a change in bowel habit. In the U.S., between 48 and 65 million people suffer from IBS, although up to 75% of patients lack a formal diagnosis (Table 1) [21,92,93]. Additionally, millions of new IBS cases are diagnosed annually, suggesting that the number of patients is increasing and more patients are now receiving a formal diagnosis [21]. Fecal calprotectin has emerged as a protein biomarker that can assist clinicians in their diagnostic evaluation of IBS. Lower levels of calprotectin can be used to distinguish IBS patients from IBD patients [94]. However, IBS can also develop in patients diagnosed with IBD, as they are defined as independent conditions [95]. Furthermore, IBS shares common symptomatology with IBD leading to cases of misdiagnosis [84]. A clinical dilemma emerges when IBD patients continue to experience gastrointestinal symptoms when in remission, leaving the clinician to decipher if this is due to active disease requiring a change in treatment, or if the patient has developed IBS [96]. 

IBS patients are currently diagnosed based on the Rome criteria, with Rome IV being the latest version, consisting of a combination of symptom-based criteria, which sometimes is supplemented by diagnostic tests that must show no abnormal findings [97]. A diagnosis of IBS is formed on the basis of persistent abdominal pain, usually in the lower abdomen, that is related to defecation accompanying a change in stool frequency or form [97,98]. Most IBS patients are classified depending on the predominant stool pattern they exhibit; IBS with diarrhea (IBS-D), IBS with constipation (IBS-C), and those that suffer from both stool patterns (IBS-M) [97,99]. 

Once identified, treatment can take on a myriad of forms due to the heterogeneity of IBS, even within the same subtype. The wide scope of disease presentation necessitates a range of treatments spanning lifestyle changes to pharmacologic intervention [100,101]. Initial therapy such as dietary modification and increased physical activity may be enough to improve the condition, but for those patients that continue to encounter moderate to severe symptoms that affect quality of life, more aggressive treatments may become necessary [100,101]. Depending on the type of IBS identified, pharmacologic options vary and can include antispasmodics, neuromodulators, and even low-dose antidepressants [102]. Costs for these treatments range from the least expensive medications totaling ~$1000 per year to treatments that cost in excess of $10,000 per year such as selective serotonin reuptake inhibitors [103].

Similar to IBD, IBS results in large disruptions both in professional productivity and personal quality of life. Annual indirect costs due to lost productivity for an IBS patient range from $1000 to over $8000 annually [9]. Reduction in quality of life for patients suffering from IBS is so profound that one study documented that patients would sacrifice 10–15 years of life expectancy for an immediate cure [104]. Diagnosing IBS accurately, through minimally invasive methods and identifying optimal treatment, could play an important role in reducing both the societal and economic burden of this syndrome [92].

### 2.6. Fibromyalgia Syndrome (FMS)

FMS is a disorder characterized by musculoskeletal pain for at least three months in all four quadrants (right and left side of the body, above and below the waist) that affects tendons, ligaments, and muscles [105]. The number of patients in the U.S. suffering from FMS has been estimated to be in excess of 6 million [20]. Each year, 5.8 million new patients are diagnosed, suggesting an increasing burden of FMS on the general population (Table 1) [24]. Common symptoms also include fatigue, sleep disturbance, morning stiffness, cognitive problems, paresthesia, headache, IBS, and anxiety [24,105].

Clinically, FMS onset is idiopathic and is not explained by some other rheumatic or systemic dysfunction. Diagnosis of FMS is based on clinical presentation and relies primarily on patient symptoms, clinical history, and physical examination [105,106]. FMS is diagnosed through exclusion once other differential diagnoses, including RA and systemic lupus erythematosus (SLE), have been considered and excluded as the underlying cause of chronic pain. No single objective test exists for FMS, resulting in diagnostic challenges to correctly identify this syndrome [106,107]. The complex nature of the diagnostic process is underscored by published data finding that FMS is both underdiagnosed 20% of the time or over-diagnosed at an astonishing rate of 60%–75% [28,108,109]. Similar to the symptoms present across both IBD and IBS, FMS shares generalized symptoms that closely resemble RA or SLE [110]. Thus, FMS normally falls within the rheumatology specialty as a condition, but patients often consult with physicians from a variety of specialties including primary care, neurology, and psychiatry [111]. On average, a diagnosis of FMS takes 2.3 years after the onset of symptoms, and patients will consult, on average, with 3.7 physicians before receiving a diagnosis [112]. In one survey, 85% of physicians across specialties (primary care physician, rheumatologist, neurologist, psychiatrist, or pain specialist) found it difficult to distinguish FMS from other conditions and 75% were not always comfortable with diagnosing the condition. This survey included 1622 physicians from eight countries with an average of 20 years of clinical experience [111].

Effective care for FMS requires a proactive patient-centric approach that provides the best chance for continued treatment adherence and condition management. Current treatment recommendations encompass both pharmacological agents as well as lifestyle changes to decrease pain and improve function [113]. While medical guidelines recommend against the use of long-term opioids, they continue to be commonly prescribed to FMS patients. The rates of opioid use in FMS ranges from 11% to 69%, and most patients will likely never accept guideline-recommended medication after initially receiving opioids, presumably due to patient resistance to alternative treatments [114,115]. This results in an unnecessary exposure of patients to this class of drug, and worst of all, addiction. Indeed, long-term opioid use in FMS has been associated with poorer outcomes than in individuals who are never prescribed opioids [114,116]. A decline in quality of life due to development of FMS is difficult enough, but needless exposure to ill-advised pain medication as well as exposure to a high-risk probability of addiction emphasizes the importance of correctly identifying FMS and initiating an appropriate treatment plan as soon as possible.

Like many functional disorders, FMS negatively impacts personal relationships, social support systems, and careers. FMS patient populations have strained mental health with higher documented levels of depression, anxiety, and lower overall physical health compared to the general population [117]. FMS patients report physical limitations that are similar to those reported by patients with osteoarthritis or RA, leading to reduced work productivity and forcing some to leave the workforce entirely [118,119,120]. Positive coping methods such as meditation, exercise, talking to friend or family member, and getting involved in hobbies can help alleviate the downturn in patient quality of life. Talking to a friend or family member and exercise have been identified as the top activities to positively cope with ongoing FMS [117].

### 2.7. Comorbidities

Comorbidities present serious expensive complications for patients diagnosed with inflammatory disease and amplify the overall direct, as well as indirect, costs associated with managing progressive illness [45]. One study found that if the initial disease could have been avoided, in turn averting associated comorbidities, savings of more than 1.15 times the average cost of the primary disease are possible [121]. While it is difficult to argue that inflammatory diseases can be avoided entirely, this study highlights potential opportunities for improved outcomes as well as reductions in healthcare costs that are achievable if early actions are taken to address inflammatory diseases and reduce the occurrence or severity of associated comorbidities. 

As stated previously, the described inflammatory diseases can share similar comorbidities. In particular, mental and mood disorders are frequently found in these populations. MS, IBD, and RA patients exhibit an increased propensity to develop anxiety and depression [44,122,123]. Depression negatively impacts therapy adherence and increases the likelihood that patients will experience serious clinical events including a relapse or flare that may result in hospitalization [124]. Additionally, all three inflammatory diseases share an increased prevalence of cardiovascular comorbidities, with RA displaying the highest prevalence [4,44,125]. In fact, heightened mortality rates associated with RA are predominantly due to cardiovascular complications with the risk of developing cardiovascular disease increasing almost two-fold, equating to a risk magnitude similar to diabetes mellitus [4,126]. The described syndromes can also become comorbidities in inflammatory diseases and are often diagnosed in the same patient. For instance, IBD/IBS have prevalence rates in the MS population that are 20% higher than the general population [2,44,86]. Similarly, FMS is observed in up to 25% of the RA population [87,88,89,90]. 

### 2.8. Clinical Benefits of Early Diagnosis

Timely diagnosis followed by immediate intervention can help stabilize disease, induce remission, minimize irreparable tissue damage, and protect against the mortality and morbidity associated with many chronic inflammatory diseases [3,11,12]. The untapped potential for better patient outcomes, in addition to reduced healthcare costs for inflammatory diseases and related comorbidities, rests on the ability to quickly and accurately diagnose patients.

### 2.9. Multiple Sclerosis

In the case of MS, early recognition of the disease is imperative because irreversible damage to the brain and spinal cord caused by subclinical disease activity has occurred prior to clinical manifestation [3]. Using current diagnostic gold standards, even patients with an early diagnosis have already experienced some level of damage to the CNS [36]. In the absence of a cure for MS, treatments seek to reduce subclinical disease activity, preserve brain volume, and slow or prevent disability progression [3]. Current treatment options are unable to reverse neurological damage that has already accumulated. However, studies have shown that early treatment with DMTs delayed progression of clinically definite multiple sclerosis and reduced the frequency of new lesions [127,128,129]. For example, changes in lesion volume were significantly improved in MS patients that received early interferon treatment. At 18 months, there were 58% fewer new or enlarging lesions and 71% fewer gadolinium-enhanced lesions in patients treated with early interferon therapy [130,131]. 

### 2.10. Rheumatoid Arthritis

Similarly, early RA diagnosis facilitates timely treatment and better overall prognoses. Within two decades of onset, 90% of patients with RA have some form of disability [132]. Of the patients treated with an aggressive management approach, utilizing frequent review and treatment escalation, 65% achieved clinical remission (absence of joint inflammation) compared to 10%–20% of patients who were treated less intensively [133]. Furthermore, intensive reduction of inflammation normalizes cardiovascular risk, which is significantly higher in patients with rheumatoid arthritis. Methotrexate, the anchor therapy in the management of RA, can reduce cardiovascular morbidity and mortality by 15%–85% [134,135]. 

### 2.11. Inflammatory Bowel Disease

Treatment of IBD seeks to achieve clinical remission and allow mucosal healing of the GI tract [136]. Early aggressive treatment is 24% more effective at achieving remission without corticosteroids and without surgical resection compared to patients receiving delayed conventional treatment [137]. Treatment of IBD and associated comorbidities can lead to polypharmacy increasing risk of detrimental drug interactions [138]. The development of comorbidities can lead to a reduction in the number of treatment options for IBD. Some IBD drugs can trigger or exacerbate concomitant disease. For example, steroid-based therapy can affect diabetes, anti-tumor necrosis factor (TNF) antibody treatment can trigger heart failure, and certain immunomodulators have been linked to cancer development [138].

While early diagnosis and treatment are frequently the recommended approach for treating each of these inflammatory conditions, even expert clinicians have difficulties pinpointing inflammatory disease in the earliest stages [84,111,139,140]. 

### 2.12. Diagnostic Delay

For medical professionals, a challenging aspect of reaching an early definitive diagnosis of an inflammatory disease is the time that accrues in the diagnostic process [141,142]. Initial development of disease is difficult to identify due to frequent delays in patients seeking medical attention, as well as the presentation of largely non-specific symptoms that disguise the underlying disease [141,142,143]. The time delay between symptom onset to diagnosis spans the length of time the patient endures discomfort due to the condition prior to seeking medical attention (medical encounter lag time), as well as the length of time it takes the physician(s) to make the diagnosis (diagnosis lag time) [142]. Primary care physicians are typically the first point of contact, but patients will undoubtedly be evaluated by multiple specialists across multiple visits to rule in or rule out an inflammatory condition. In many cases, patients will be evaluated by three or more physicians prior to obtaining a diagnosis [112,144,145].

The changing landscape of health insurance in the last decade has further elongated diagnostic delay. The rapid expansion of high-deductible health plans (HDHP) has had a noticeable effect on patient utilization of healthcare [146,147]. At least one study has shown that HDHP members experience close to a 10% reduction in use of doctor office visits [148]. Patients are delaying or avoiding what they perceive to be unnecessary visits as their main strategy for limiting costs [149]. Medical encounter lag time leads to poorer outcomes for patients suffering from inflammatory conditions as the underlying damage inevitably accumulates. 

One of the most prominent diseases where both medical encounter and diagnosis lag time occurs is MS. Due to the slow initial development of disease, patients typically do not seek physician assistance immediately and a diagnosis of clinically definite MS is delayed [144]. The McDonald criteria emphasizes the need to demonstrate dissemination of brain lesions in space and time on MRI results. Typically, to fulfill the requirement of time, a second follow-up MRI must be performed following the baseline scan. This second scan can occur days to months following the initial scan [36,37]. The median time from MS suggestive symptoms to a referral to a specialist or the initiation of treatment can be greater than two years and, in complicated cases, up to seven years [46,144,150].

IBD and IBS patients also experience diagnostic delays lasting as long as five years [10,151,152]. In pain-associated symptomology, RA and FMS have reported median lag times from symptom initiation to diagnosis of up to two years [112,143]. The diagnostic delays that accompany these inflammatory conditions underscore the need for additional approaches and tools capable of providing actionable accurate information quickly. 

### 2.13. Danger of Misdiagnosis

Misdiagnosis of inflammatory conditions can have serious implications for both patient and physician. Patients treated with unnecessary medications may experience adverse events from therapy, attending physicians may face the threat of legal action, and already stretched healthcare systems shoulder the financial burden of treatments that can cost tens of thousands of dollars per year [153]. Given the inherently unpredictable and varying clinical presentation of these conditions, physicians are faced with the difficult task of identifying and diagnosing them quickly and correctly. Continued innovation and commercialization of tools to supplement current diagnostic testing is essential to provide physicians with the best information to minimize misdiagnosis.

The challenge of misdiagnosis extends to all inflammatory and related conditions. In particular, misdiagnosis of MS has been most prominently analyzed in contemporary clinical practice, and surveys have found that patients receive incorrect diagnoses in up to 10% of cases (Table 1) [25,140]. Studies also indicate that of the total referrals to MS subspecialty centers, 30%–67% were ultimately determined not to have MS [140]. Alternative diagnoses, such as functional neurologic disorders, migraine, vascular disease, as well as uncommon inflammatory, infectious, and metabolic disorders, may mimic MS. A common error is to over-interpret multiple hyper-intense lesions apparent on MRIs as equivalent to MS, while clinical symptoms consistent with MS diagnosis are absent [154]. Furthermore, in atypical and challenging cases, patient history and clinical examination findings accompanied only by abnormal brain MRIs may not be sufficient [25]. In these instances, it is not uncommon for misdiagnosed patients to be treated with DMTs. A multicenter study revealed that 50% of patients who had been incorrectly diagnosed with MS carried the misdiagnosis for at least 3 years, and more than 5% were misdiagnosed for over 20 years. In the same study, 31% incurred unnecessary morbidity as a direct result of misdiagnosis [140,155]. To avoid misdiagnosis, when applying the McDonald criteria, it is necessary to consider alternative diagnoses even when symptoms are typical for MS [25,140]. Defining signs, symptoms, and ‘red flags’ that increase or decrease diagnostic confidence in a diagnosis of MS versus other conditions remains an area of active investigation [156]. These initiatives have led to the creation of new approaches that include novel laboratory tests using cerebral spinal fluid, determination of RNA expression patterns in peripheral whole blood, and new technologies to analyze radiographic imaging information. Each of these technologies could add important new information to the neurologist’s diagnostic armamentarium [38,140].

Diagnosing disease during the earliest stages of development is challenging, and tools to definitively diagnose inflammatory diseases early are not yet available. In cases of early diagnosis, there is a necessity for the physician to re-evaluate the patient during the course of disease to confirm that the initial diagnosis is correct. Patients unresponsive to initial treatment must be re-examined with differential diagnoses in mind, as sometimes the wrong diagnosis may be the reason for poor response to treatment [157]. For example, a rare chronic infection, Whipple’s disease (WD), may present with predominant joint manifestations (including radiologic evidence of erosive joint destruction) that mimics RA [158]. In this instance, a misdiagnosis of RA and initiation of therapy would prove ineffective and dangerous to the patient. The typical treatment for WD, an antibiotic regime, differs greatly from an immunosuppressive treatment that is typically utilized for RA. An immunosuppressive treatment course prescribed in error due to a misdiagnosis of RA may accelerate progression of WD and even prove fatal [159]. A recent review of seven clinically misdiagnosed patients suffering from WD found that six received immunosuppressive therapy consisting of synthetic as well as biologic disease modifying anti-rheumatic drugs for a median of 15 months prior to discontinuation [27]. These cases highlight the importance of clinical follow-up, especially when diagnosis is made early in disease development.

Disease mimics that are often misidentified as inflammatory conditions add an additional layer of complexity for clinicians. Recognition among practicing physicians of the difficulty in utilizing general signs and symptoms for diagnosis of inflammatory conditions, especially in the early stages of development, will hopefully push for development of better tools to alleviate time delays that add to the diagnostic odyssey for patients [111,152]. Campaigns to promote awareness among practicing ‘front line’ clinicians, including those in primary care, could alleviate some of the time delay and increase the speed in addition to accuracy of diagnosis [3].

## 3. Estimating Financial Burden and Potential Sources of Healthcare Cost Savings

Beyond the clinical burden, inflammatory diseases and related conditions incur significant financial cost across the entire healthcare continuum from patients to payers. Unlike acute events, the chronic nature of inflammatory diseases and related conditions results in a financial burden that all parties face for the remainder of a patient’s life. Although treatment is improving, there is no absolute cure for these conditions. However, early diagnosis and treatment can have a profound impact on both short- and long-term clinical outcomes [127,128,129,133,136]. The following summarizes an effort to quantify the financial impact, investigate cost drivers, and calculate how improved clinical efficiencies for diagnosis and treatment of inflammatory diseases and chronic syndromes can reduce overall financial burden. 

### 3.1. Cost Savings Estimate Equation Development

A review of the U.S. healthcare costs associated with inflammatory disease and related conditions resulted in the identification of four fundamental factors that significantly impact overall direct healthcare costs. This analysis specifically focused on understanding potential annual savings that could be obtained across inflammatory diseases and related chronic syndromes through (1) managing treatment costs, (2) controlling condition progression, (3) avoiding high cost events, and (4) promoting treatment adherence. Each of these four factors provide a comprehensive description of the overall direct costs and play pivotal roles in realizing potential savings. Furthermore, indirect costs associated with the patient’s journey were not included in the calculation.

**Figure 1:**
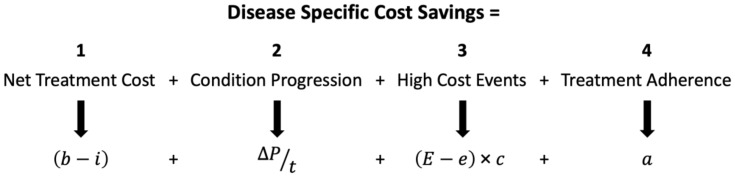
Equation for disease specific cost-savings estimate. Variable definitions by factor. Factor 1: *b* = all direct costs without condition specific treatment; *i* = all direct costs with a range from condition specific average treatment cost (*i*_1_) to cost-efficient treatment (*i*_2_). Factor 2: *ΔP* = *P_H_ − P_L_*; net cost of condition progression (*P_H_ =* all direct costs when treated high severity, *P_L_* = all direct costs when treated low severity); *t* = time of condition progression. Factor 3: *E =* high-cost event probability without condition specific treatment; *e* = high-cost event probability with condition specific average treatment; *c* = average high-cost event cost. Factor 4: *a* = treatment adherence savings. The resulting condition specific cost savings are per affected patient, per year.

A cost savings equation (Figure 1) has been developed to explore the impact of each of these disease management areas on total healthcare expense per affected patient per year. By utilizing various cost ranges published in peer-reviewed literature, an estimate of potential annual cost savings can be derived.

The first factor in the equation *(b–i)* calculates the additional net cost of the condition when varying degrees of treatment are administered. This factor includes the average total cost of healthcare utilization a patient requires without undergoing condition specific treatment (variable *b*), and the average total cost including a range of treatments specific to the condition (variable *i*). The second factor *(ΔP/t)* calculates annualized increase in costs over time, *t,* as a condition progresses. The *ΔP* variable was estimated as the change in total cost from a low disease-severity cost (*P_L_*) to a high disease-severity cost (*P_H_*) that occurs over a progression time period, *t*, defined as the amount of time that occurs during the assumed progression of the condition. Due to the chronic nature of these conditions, disease progression occurs over a long period of time, measured in years. The third factor [(*E − e*) × *c*] describes the costs related to high-cost events, which are defined as events that can occur based on an average probability and result in high short-term financial costs. Event examples for each of the inflammatory diseases and related conditions of interest include: MS relapse, abrupt gastrointestinal surgery in the case of IBD, joint replacement due to RA, and addiction recovery from pharmaceuticals during FMS treatment [35,68,160,161]. The variable *E* is defined as the high-cost event probability per patient per year if baseline treatment not specific to the condition is administered. The variable *e* is the high-cost event probability per patient per year if average treatment for the condition is administered. The difference between the probability of baseline high costs events (*E*) with baseline treatment and the probability of high-cost events with average treatment (*e*) is multiplied by the one-time average cost of the high-cost event (variable *c*). The fourth and final factor represents the impact of greater patient treatment adherence (variable *a*). Variable *a* is a literature-cited estimate of how high levels of treatment adherence results in a net cost/savings via potential reduced total healthcare utilization (physician visits, ER admission, etc.). The factors added together culminate in a net savings/cost that is an annual estimate per affected patient. This result provides insights into each disease’s cost profile and how early accurate diagnosis and treatment may affect that profile. 

### 3.2. Equation Assumptions, Data Availability, and Limitations

While developing this equation, assumptions were made in order for the analysis to be compatible with various conditions, as well as the data resources available. First, after a broad review of relevant peer-reviewed literature, the cost factors selected were assumed to represent treatments that were shown to have a positive effect on clinical outcomes. Secondly, it was assumed that, on average, inflammatory diseases worsen over time and deteriorate more rapidly without treatment [162]. Additionally, this deterioration directly correlates with increased healthcare costs [41,163,164,165]. With respect to treatment adherence, after the earliest possible diagnosis is made, we assumed clinicians would be able to offer treatment options that exhibit a more tolerable side effect and risk profile to patients [42,65,79]. In these scenarios, it was assumed that patients are incentivized to delay or arrest disease progression while they are most healthy, can be placed on treatments where fewer side effects have been reported, and that patients are more likely to adhere to these treatment choices [166]. Further, due to the subjective nature and inconsistent financial indirect cost estimates found during the analysis of inflammatory diseases and related conditions, the indirect costs are not included in this financial analysis. Finally, it is expected that any given population with affected patients would have a range of actual costs related to the equation factors. The inputs are averaged and annualized when actual lifetime costs of potential patients may not be linear.

### 3.3. Equation Use and Inputs

The variable inputs were selected by analyzing relevant peer-reviewed sources with data regarding the epidemiology, cost burden, interventional efficacy, and treatment outcomes for each of the inflammatory diseases and related conditions considered. Each peer-reviewed source’s data input followed the equation logic and included specific costs that tie into each equation factor. Generalized costs that were not ascribed to a specific factor were not included. Healthcare cost data for inflammatory diseases and chronic conditions, including IBS and FMS, can be sparse. No single source was able to provide the necessary information for any one disease or syndrome. Thus, the literature review conducted cross-referenced the cost factors listed in Table 2. The sources used in the financial modeling were published over the course of many years. However, approximately 50% of the cited figures and variable inputs were published between 2013 and 2018 and 90% of cited articles were published between 2008 and 2018. All costs were inflated to 2018 dollars by utilizing the US Bureau of Labor Statistics consumer price index inflation calculator.

In order to estimate a range of savings, on average or in a best-case scenario, analysis conducted using the equation in Figure 1 included changing the input for the treatment cost factor, variable *i*. To define the average treatment cost factor (variable *i* = *i*_1_), an average of all treatment costs for a specific condition was calculated using reported average costs for each individual treatment. This average is a conservative approximation of treatment cost. Additionally, the most cost-effective treatment was identified and calculated as variable *i* = *i*_2_ to provide a best-case scenario. These two calculation scenarios, average treatment (*b* − *i*_1_) and most cost-effective treatment (*b* − *i*_2_), provide a range of cost savings where variable *b* is the baseline average cost of healthcare utilization a patient requires without undergoing a condition specific treatment.

### 3.4. Input Assumptions, Data Availability, and Limitations

When identifying the proper input values for each variable, the following assumptions were made in order to normalize values across all medical conditions analyzed. When possible, the cost assumptions reflect all direct healthcare costs that are associated with each disease. This assumption included costs of associated disease or chronic syndrome comorbidities. As defined, condition progression can occur over a significant portion of a patient’s lifespan. The variable *t* was assumed to be one decade (10 years) for all inflammatory diseases and related conditions reviewed. This value provides a conservative estimate of how disease severity progresses over time given that the *ΔP* endpoints, *P_L_* and *P_H_*, are averages, which removed cost outliers. Although each disease carries risk of more rapid progression than 10 years, this analysis focused on the first 10 years of progression, which was assumed to be when early diagnosis and treatment has the greatest opportunity to positively impact patient outcomes. Lastly, several inputs presented in Table 2 are shown as not available (N/A) for IBS. Available IBS peer-reviewed cost data were published more than a decade ago and did not contain detail around various treatment effects like the other conditions reviewed. Also, IBS does not have an associated high-risk event or published treatment adherence data. Therefore, those factors were not included in the final cost savings analysis for IBS. These empty (not available) input values carry through to the rest of the tables.

### 3.5. Results and Discussion of Cost Analysis Focusing on Inflammatory Disease and Related Syndromes

The results of calculations utilizing input values identified through literature review (Table 2) are shown in Table 3 and Table 4. For all conditions and both treatment scenarios, the potential for annual savings was positive, except for the average treatment scenario for MS (variable *i* = *i*_1_).

Multiple sclerosis is an interesting case to use for the proposed equation and analysis. Starting with Factor 1, net treatment cost, the annual average total cost of an MS patient without receiving MS specific treatment is $16,301 (variable *b*) [41]. If treated with the average condition-specific treatment, annual costs rise to $41,157 (variable *i* = *i*_1_). However, the most cost-effective condition-specific treatment reviewed costs $26,397 (variable *i* = *i*_2_) per year [41]. Thus, treating an average MS patient will result in additional cost of $10,096 ($16,301 less $26,397) to $24,856 ($16,301 less $41,157) per year. Factor 2, condition progression over time (*ΔP/t*), shows that a low-severity, treated MS patient (*P_L_*), costs $31,477 per year on average whereas a high-severity, treated patient (*P_H_*) costs $104,924 per year [41,163]. The difference between the high-severity and low-severity costs is (*ΔP*) $73,447. Since it is assumed a patient will take approximately a decade to progress from a low to high severity, this annual cost difference is averaged over *t* = 10 years, resulting in approximately $7345 of annual disease progression cost. Next, Factor 3, high-cost events ([*(E − e) × c*]), reveals that an MS relapse costs $18,177 per event (variable *c*), on average [50]. A MS patient receiving baseline therapy, without condition-specific treatment, experiences a relapse three times in four years on average, resulting in a 75% average chance of relapse each year (variable *E*) [41]. A MS patient on an average MS-specific treatment has a 61% average chance of relapse each year (variable *e*) [41]. Thus, Factor 3 results in annualized savings of $2544. Finally, Factor 4, adherence to treatment (*a*), displays that an MS patient with the highest adherence to treatment saves on average $6162 per year in non-treatment related direct costs [43]. Overall, the final value using condition-specific treatment with an average cost (*i* = *i*_1_), is a net cost increase of $8805; whereas, the final value using treatment that is the most cost-effective (*i* = *i*_2_), is a net cost savings of $5995. 

Together, these four fundamental factors and their respective individual variables estimate the cost effects of treating patients as early and as cost-effectively as possible. The average treatment scenario (*b − i*_1_) results in net savings/(*cost increase*) of: $(8805) for MS; $4385 for IBD (Crohn’s and UC); $8808 for RA; $522 for FMS; and $642 for IBS, all per affected patient on an annual basis (Table 3). The most cost-effective treatment scenario (*b − i*_2_) results in net savings of $5955 for MS; $12,706 for IBD; $11,025 for RA; and $1412 for FMS, per affected patient per year (Table 3).

Though each equation factor is clinically interrelated, evaluation of the factors separately provides insights into how early effective treatment may lead to drastic inflammatory disease cost improvement. Each factor had a varied impact on the cost savings potential for each inflammatory condition. No one factor calculated provided the greatest savings for each the conditions reviewed. For Factor 1, net treatment cost, the range from the lowest to the highest cost spanned from $539 (IBS, Table 3) to a $24,856 (MS, Table 3). Factor 2, controlled condition progression over time, was most impactful for MS equaling $7345 (46% of the net positive results) (Table 4). Overall, Factor 3, avoided high-cost events, had very little effect on RA ($251; 2%) and FMS ($9; 0%) due to the low baseline likelihood of their events, particularly compared to MS ($2544; 16%) and IBD ($3833; 25%) where high-cost events are more likely (Table 4). Lastly, Factor 4 totaled $9792 (64% of the net positive results) and $8622 (72% of the net positive results) for IBD and RA, respectively. Overall, Factor 4, promoted treatment adherence, had the most consistent positive effect ($1451–$9792; 23%–72% of the net positive results, Table 4). This influence is supported by previously published studies that showed an average overall cost savings from 15% (MS) to 65% (IBD) when patient treatment adherence was improved [43,164,181,182]. Additionally, these data indicate that efforts to ensure patient treatment plan adherence should be a high priority for physicians and payers once the most cost-effective treatment has been initiated. 

Only in the MS average treatment cost scenario (variable *i* = *i*_1_) did the cost of additional treatment result in a net cost increase. This is due to the extremely high average cost of MS treatment (i.e., DMTs) as compared with lower-cost treatment options available in other conditions [183,184]. Even though early accurate treatment can control associated costs (Factors 2, 3, and 4), the incremental cost to treat for MS (Factor 1) was higher than savings recognized. However, for the other inflammatory diseases and related conditions, as well as in the MS most cost-effective treatment scenario, the average savings that accumulate as a result from reduced disease progression over time, reduced risk of high-cost events, and higher treatment adherence, were significantly more than the average cost to treat. 

Misdiagnosis of these inflammatory diseases and chronic syndromes is not uncommon, resulting in significant unnecessary expense. For example, if one MS patient is misdiagnosed and treated, this patient could accrue costs that equate to the average annual spend for an MS patient, $41,157 (Table 2) [41]. Because the annual misdiagnosis rate for MS is 10% annually (Table 1), for every 10 MS members in a population, there is risk of at least $41,157 of wasted spend [25]. Similar logic can be applied across the other inflammatory diseases and related conditions reviewed.

Early diagnosis and treatment have positive, compelling effects on indirect expenses. In addition to direct medical costs associated with inflammatory disease and related conditions, lost working days (absenteeism) and minimal productivity while on the job (presenteeism) amount to significant indirect costs. In addition to the above review and analysis of direct costs, average indirect costs range from $6448 (IBD) to $32,179 (MS) per member per year without treatment [41,164]. When treated, these indirect costs can also be controlled. Treated patients average indirect costs ranged from $4467 (IBD) to $25,802 (MS) [41,164]. Indirect cost savings due to treatment can range from $1099 (FMS) to $6377 (MS), depending on the condition [41,170].

Overall, this means early and appropriate management of an inflammatory condition, via the outlined fundamental factors, controls and minimizes the associated direct healthcare spend. By investing in early diagnosis and treatment (Factor 1), the healthcare system can financially benefit from controlled condition progression (Factor 2), avoided high cost-events (Factor 3), and enhanced adherence to treatment plans (Factor 4). In particular, the range of savings shown by changing the course of treatment from average to cost-efficient options highlights the importance of this factor and how it relates to the subsequent factors.

## 4. Conclusions

A long sought-after goal across a wide range of healthcare specialties is to devise strategies that enhance patient care and simultaneously reduce expenses. Inflammatory diseases and chronic syndromes currently impose an immense social and economic burden on society in terms of both direct and indirect healthcare costs. While published annual direct costs attributable to the reviewed group of inflammatory diseases and syndromes is currently greater than $90B ($35B MS, IBD, RA and $55B IBS, FMS), this estimation understates the true cost potential. As healthcare diagnostics continue to improve, more undiagnosed patients will be properly diagnosed and treated, and misdiagnosis rates will decrease. It is conceivable that when the total cost of these conditions is calculated by multiplying the selected per patient direct cost averages in (Table 2) by national prevalence in (Table 1), this group of conditions may cost upwards of $300B annually, heavily influenced by the effect of FMS and IBS. Thus, clinical strategies to curtail these direct costs are of paramount importance. 

In the case of inflammatory disease, their incurable progressive nature results in unavoidable lifetime expense. Over the past decade, we have witnessed the revision of diagnostic criteria to facilitate earlier detection and faster treatment, particularly in the field of multiple sclerosis [185]. While each inflammatory disease exhibits a diverse pathophysiology and clinical phenotype, the treatments that are available are highly effective at controlling progression of disease. Early immediate action can make an immense impact on short- and long-term clinical outcomes and lifetime healthcare costs. 

Inflammatory diseases and related chronic syndromes incur a wide-range of costs depending on the severity of disease, number of high-cost events, therapies utilized, and treatment adherence rates. In the face of a substantial impact upon society and the economy, these factors represent significant opportunities to effect positive outcomes. When compared to more publicized diseases, such as cancer and chronic obstructive pulmonary disease (COPD), inflammatory diseases have a greater disease prevalence and are just as costly. Taken together, all inflammatory diseases (>80) affect up to 50M Americans according to the American Autoimmune Related Diseases Association (AARDA) and the annual direct healthcare costs exceed $100B. In contrast, cancer affects 15M Americans at a cost of $125B while COPD affects up to 24M Americans with an annual cost of $50B [186,187]. Similar to efforts that are currently underway in cancer and COPD research, programs are needed to develop novel approaches to reduce the high costs associated with inflammatory diseases, related chronic syndromes, and associated comorbidities. The healthcare community needs to direct its energies to ensure that early accurate disease diagnoses are achieved, the most cost-effective treatments are prescribed, and programs supporting patient treatment adherence are developed. 

Affecting patients regardless of gender, age, ethnicity, geography, and socioeconomic status, inflammatory diseases and related chronic syndromes are not confined to specific areas or groups of people in the U.S. All geographic regions and socioeconomic classes are impacted. Inflammatory diseases have been likened to an ‘invisible epidemic’ [188]. Our work denotes specific points in the patient journey that can be exploited to curb the negative clinical and economic outcomes of these conditions. It is our hope that the information presented here helps garner the attention this group of conditions deserves by promoting increased patient advocacy efforts and research funding to address this important area of human health and disease.

## Figures and Tables

**Table 1:** General population statistics across indicated inflammatory diseases and syndromes.

	Inflammatory Diseases						Chronic Syndromes			
Statistic Description	MS		IBD (UC + CD)		RA		FMS		IBS	
		Ref.		Ref.		Ref.		Ref.		Ref.
Disease Prevalence	0.3%	[15]	0.5%	[18]	0.5%	[19]	2.0%	[20]	15.0%	[21]
Disease Incidence	0.005%	[17]	0.02%	[22]	0.04%	[23]	1.8%	[24]	6.7%	[21]
Average Misdiagnosis	10.0%	[25]	23.5%	[26]	0.1%	[27]	75.0%	[28]	11%	[29]

Abbreviations: MS = multiple sclerosis; IBD (UC + CD) = inflammatory bowel disease (ulcerative colitis, Crohn’s disease); RA = rheumatoid arthritis; FMS = fibromyalgia syndrome; IBS = irritable bowel syndrome. Literature sources are cited next to each value.

**Table 2:** Direct costs inputs to financial analysis equation.

		Inflammatory Diseases						Chronic Syndromes			
Direct Costs Variable Inputs		MS		IBD (UC + CD)		RA		FMS		IBS	
			Ref.		Ref.		Ref.		Ref.		Ref.
All direct costs without condition specific treatment (avg.)	*b*	$16,301	[41]	$7570	[167,168]	$10,060	[169]	$4809	[170]	$4565	[171]
All direct costs with condition specific average treatment (avg.)	*i*_1_	$41,157	[41]	$18,512	[164]	$13,255	[172]	$6239	[20]	$5103	[171]
All direct costs with most cost effective condition specific treatment (avg.)	*i*_2_	$26,397	[41]	$10,191	[173]	$11,038	[174]	$5349	[20]	N/A	
All direct costs when treated—Low Severity (avg.)	*P_L_*	$31,477	[41,163]	$14,046	[164]	$4430	[165]	$5349	[20]	$490	[175]
All direct costs when treated—High Severity (avg.)	*P_H_*	$104,924	[41,163]	$31,060	[164]	$35,695	[165]	$10,269	[20]	$12,999	[175]
Incremental disease progression ‡	*ΔP*	$73,447	*	$17,014	*	$31,498	*	$4920	*	$11,809	*
Assumed disease progression years †	*t*	10	†	10	†	10		10	†	10	†
Adverse event probability without condition specific treatment (avg.)	*E*	75.00%	[41]	60.00%	[164]	2.00%	[176]	0.78%	[177]	N/A	
Adverse event cost (avg.)	*c*	$18,177	[50]	$38,333	[164]	$25,173	[178]	$23,018	[177]	N/A	
Adverse event probability with average condition specific treatment (avg.)	*e*	61.00%	[41]	50.00%	[179]	1.00%	[180]	0.74%	[177]	N/A	
Treatment adherence savings (avg.)	*a*	$6162	[43]	$9792	[164]	$8622	[181]	$1451	[182]	N/A	

Abbreviations: “Avg.” = average. * Indicates calculated value; † assumed decade of disease progression; ‡ calculated as cost difference between high and low severity. Literature sources are cited next to each value. N/A = literature source unavailable.

**Table 3:** Results of financial analysis equation for average and most cost-effective treatment scenarios.

	Inflammatory Diseases			Chronic Syndromes	
Direct Costs Calculation Results	MS	IBD (UC + CD)	RA	FMS	IBS
Factor 1 − Average scenario (*b* − *i*_1_)	$(24,856)	$(10,942)	$(3195)	$(1430)	$(539)
Factor 1 − Best case Scenario (*b* − *i*_2_)	$(10,096)	$(2621)	$(978)	$(540)	N/A
Factor 2 (ΔP/t)	$7345	$1701	$3129	$492	$1181
Factor 3 (*E* − *e*) × *c*	$2544	$3833	$251	$9	N/A
Factor 4 (*a*)	$6162	$9792	$8622	$1451	N/A
**Calc. Result − Average scenario (*i* = *i*_1_)**	**$(8805)**	**$4385**	**$8808**	**$522**	**$642**
**Calc. Result − Best case scenario (*i* = *i*_2_)**	**$5955**	**$12,706**	**$11,025**	**$1412**	**N/A**

Net savings for each factor are presented as a positive number. Net cost increases are presented as a (negative) number. N/A = literature source for calculation unavailable.

**Table 4:** Results of financial analysis equation with net positive savings factors totals.

	Inflammatory Diseases						Chronic Syndromes			
Direct Costs Calculation Results	MS		IBD (UC + CD)		RA		FMS		IBS	
Savings Factors	$	% of total	$	% of total	$	% of total	$	% of total	$	% of total
Factor 2 (ΔP/t)	$7345	46%	$1701	11%	$3129	26%	$492	25%	$1181	100%
Factor 3 (*E* − *e*) × *c*	$2544	16%	$3833	25%	$251	2%	$9	0%	N/A	N/A
Factor 4 (*a*)	$6162	38%	$9792	64%	$8622	72%	$1451	75%	N/A	N/A
**Total Potential Savings**	**$16,051**		**$15,326**		**$12,002**		**$1952**		**$1181**	

N/A = literature source for calculation unavailable.
